# The N-Terminal HSDCIF Motif Is Required for Cell Surface Trafficking and Dimerization of Family B G Protein Coupled Receptor PAC1

**DOI:** 10.1371/journal.pone.0051811

**Published:** 2012-12-21

**Authors:** Rongjie Yu, Xiaoling Guo, Jiaping Zhong, Mei Li, Zhixing Zeng, Huahua Zhang

**Affiliations:** 1 Cell Biology Institute, the Department of Cell Biology, Jinan University, Guangzhou, China; 2 Laboratory of Medical Genetics of Guangdong Medical College, Dongguan, Guangdong, China; University of Rouen, France

## Abstract

PAC1 is PACAP (pituitary adenylate cyclase-activating polypeptide) preferring receptor belonging to class B G protein coupled receptor (GPCR) mediating the most effects of PACAP. The important role of G protein coupled receptor homo/heteromerization in receptor folding, maturation, trafficking, and cell surface expression has become increasingly evident. The bimolecular fluorescence complementation (BiFC) and bioluminescence resonance energy transfer (BRET) assay were used in this research to confirm the dimerization of PAC1 for the first time. The structure-activity relationship focused on the N-terminal HSDCIF motif, which locates behind the signal sequence and has high homology with PACAP (1–6), was assayed using a receptor mutant with the deletion of the HSDCIF motif. The fluorescence confocal microscope observation showed that the deletion of the HSDCIF motif impaired the cell delivery of PAC1. The results of BiFC, BRET and westernblot indicated that the deletion of HSDCIF motif and the replacement of the Cys residue with Ala in HSDCIF motif resulted in the disruption of receptor dimerization. And the exogenous chemically synthesized oligopeptide HSDCIF (100 nmol/L) not only down-regulated the dimerization of PAC1, induced the internalization of PAC1, but also inhibited the proliferation of CHO cells expressing PAC1 stably and decreased the activity of PACAP on the cell viability. All these data suggested that the N-terminal HSDCIF motif played key role in the trafficking and the dimerization of PAC1, and the exogenous oligopeptide HSDCIF had effects on the cell signaling, trafficking and the dimerization of PAC1.

## Introduction

The neuropeptide pituitary adenylate cyclaseactivating polypeptide (PACAP) is a member of the vasoactive intestinal polypeptide (VIP)/secretin growth hormone /eleasing hormone/glucagon superfamily, which also includes secretin, VIP, glucagon-like peptide 1 (GLP-1) and glucose-dependent insulinotropic peptide (GIP) [1]. PACAP elicits various biological actions via three types of G-protein–linked receptors, a PACAP-preferring (PAC1) and VIP-shared (VPAC1 and VPAC2) receptors termed according to their relative affinity for PACAP and VIP [1], [2]. PAC1 receptor has a much higher affinity for PACAP than for VIP, whereas VPAC receptors recognize all peptides with similar high affinity [2].

**Figure 1:**
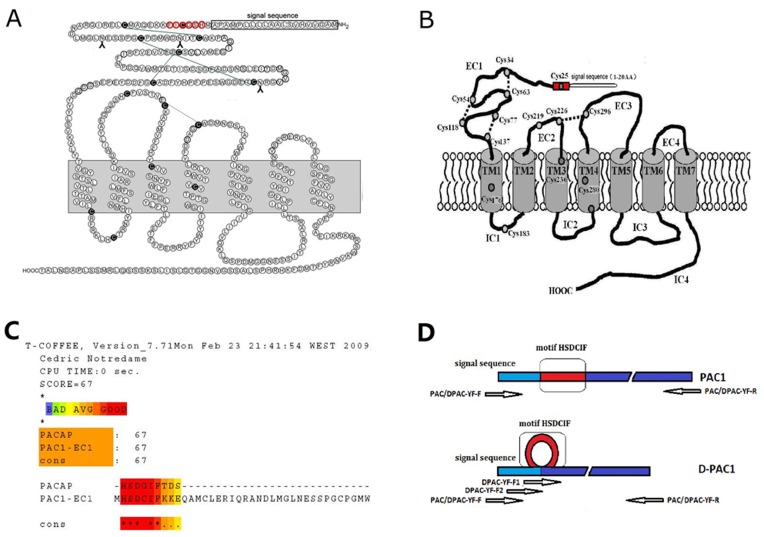
The structure sketch map of PAC1 (A, B). The HSDCIF motif is indicated as red. The homology analysis between PACAP and PAC1-EC1 (C). The red region showed the HSDCIF motif and its high homologue PACAP (1–6). The construction of D-PAC1 (D). The gene coding D-PAC1 with the deletion of the HSDCIF motif was amplified using over-lap PCR (The arrows represented the primers used to amplify the genes coding D-PAC1 and PAC1.).

PAC1 and VPACs all belong to class B G protein-coupled receptors (GPCR) family, which are endogenously activated by large peptide hormones, including members of the secretin/glucagon/GHRH superfamily, parathyroid hormone (PTH), corticotropin-releasing factor (CRF) and members of the calcitonin family [3], [4], [5]. Class B is a small family of GPCRs with low sequence and structural homologies with other members of the GPCRs superfamily. In common with other members of class B GPCRs, PAC1 shows a specific structural feature; a large extracellular N-terminal domain that contains several conserved amino acids, such as six cysteines, a hydrophobic N-terminal signal peptide and several potential N-glycosylation sites, both of which are considered crucial for receptor conformation and ligand interaction [3] (shown in Fig. 1A, B). Recently, it has been shown that the dimerization or oligomerization of GPCR may affect the physiological and pharmacological profiles of GPCR, such as trafficking of newly synthesized receptors to the cell surface, allosteric modulation of ligand binding, signaling specificity, co-internalization, or cross-inhibition of GPCRs. [6], [7], [8], [9]. Furthermore the secretin receptor, VPAC1 and VPAC2, which also are members of class B GPCR and have close relationship with PAC1, have been reported to form oligomerization (homo- or hetero-) [10], [11], [12]. We hypothesized that PAC1 can form dimer or oligomer, but till now, there is no direct proof on the dimerization or the oligomerization of PAC1. In this research, we used the techniques: bioluminescence resonance energy transfer (BRET) and bimolecular fluorescence complementation (BiFC), which have been accepted in the detection of oligomerization of GPCR [13], to verify the dimerization of PAC1.

Our previous report has indicated that the N-terminal extracellular domain, also called the first extracellular (EC1) domain of PAC1, has a motif consist of six amino acid residue His-Ser-Asp-Cys-Ile-Phe (HSDCIF) (indicated in red in Fig. 1A, B), which locates just behind the putative signal sequence, has a very high homology with PACAP (1–6) (HSDGIF) [14]. There is only one residue different (Cys vs.Gly) between the HSDCIF motif of PAC1 and PACAP (1–6) (HSDGIF), which is responsible for the activation of PAC1 (Fig. 1C) [15], [16]. And this Cys residue is not be included in the conserved six cysteines of the extracellular N-terminal domain of class B GPCR (as shown in Fig. 1A, B) and is not reported to form the known disulfide bond. The structure-activity relationship focused on this HSDCIF motif attracted our great interest. In this research, a mutant PAC1 with the deletion of the HSDCIF motif (named D-PAC1) and a mutant PAC1 with the replacement of Cys in the HSDCIF motif with Ala (named M-PAC1) were constructed and expressed in Chinese hamster ovary (CHO) cells. By this way, we wanted to detect the role of this motif and its effects on the properties of PAC1, such as cell surface delivery and dimerization.

Compared with the intact PAC1, the deletion mutant of PAC1 (D-PAC1) not only lost the ability of trafficking to the cell surface, but also lost the ability to form dimer. The exogenous chemically synthesized oligopeptide HSDCIF induced the internalization of intact PAC1, disrupted the dimerization of intact PAC1 and inhibited the viability of the CHO cells expressing PAC1 stably. It was also found that M-PAC1 was trafficked to the cell membrane normally but failed to form dimer, which indicated that the Cys in the HSDCIF motif played a key role in the dimerization of PAC1.

## Materials and Methods

### Materials

All materials for cell culture and transfection reagents were from Invitrogen (Carlsbad, CA). Reagents for molecular biological techniques were obtained from Takara (Dalian, China) and QIAGEN (Valencia, CA). The oligopeptide HSDCIF and PACAP27 were synthesized by Qiangrao Biological Company (Shanghai, China). cDNA encoding mouse PAC1 (Normal/Hop) isoform (splice variant with no deletion in EC1 and with a hop insertion in the third intracellular cytoplasmic (IC3) loop [17] ) was from GeneCopoeia agented by the Funeng Gene Company (Guangzhou, China). The eukaryotic expression vectors pEYFP-N (containing the gene encoding yellow fluorescent protein (YFP)), pRluc-N (containing the gene encoding Renilla luciferase (Rlu)) were purchased from Yingrun Biological Company (Changsha, China).

### Plasmids and Mutagenesis

The gene encoding the deletion mutant PAC1 with the deletion of the HSDCIF motif named D-PAC1 was amplified by three-step PCR using the overlap primers as shown in Fig. 1 D and Tab. 1. The gene encoding Cys mutant PAC1 (named M-PAC1) with the replacement of the Cys in HSDCIF motif with Ala was also amplified by two-step PCR using the overlap primers as shown in Tab. 1. For BRET studies, the intact PAC1 gene, the D-PAC1 gene and the M-PAC1 gene were cloned into the vectors pEYFP-N and pRluc-N respectively to construct the recombinant expression vectors PAC-YFP, D-PAC-YFP, M-PAC-YFP, PAC-Rlu, D-PAC-Rlu and M-PAC-Rlu which made the receptors tagged at the carboxyl terminus with either YFP (using as donor) or Rlu (using as acceptor). For BiFC studies, the sequence encoding the N-terminal 172 amino acid residues of YFP was amplify and added with the TAA termination codon by PCR, and was subcloned into the recombinant expression vectors PAC-YFP, D-PAC-YFP and M-PAC-YFP replacing the intact YFP gene to produce expression vectors PAC-Y/N, D-PAC-Y/N and M-PAC-Y/N, which allowed the receptors tagged at the carboxyl terminus with the N-terminal 172 amino acid residues of YFP. And the recombinant vectors PAC-Y/C, D-PAC-Y/C and M-PAC-Y/C, which made the receptors tagged at the carboxyl terminus with the C-terminal 67 amino acid residues of YFP were constructed in the same way. The primers for the all the constructions were in Tab. 1.

**Table 1:** Oligonucleotide primers employed.

Primer	Sequence 5′–3′	Purpose
DPAC-YF-F1DPAC-YF-F2	CTGCTGCCTATGGCTATTGCTATGAAGAAGGAGCAAGCCATGTGCCTGGAG CTGCAGCTCTCCCTGACTGCTCTCCTCCTGCTGCCTATGGCTATTGCTATG	Amplification of the gene encoding D-PAC1 by overlap PCR (as shown in Fig. 1C)
MPAC-YF-F1	TG ACT GCT CTC CTC CTG CTG CCT ATG GCT ATT GCT ATG CAC TCT GAC *GCG* ATC TTC AAG	Amplification of the gene encoding M-PAC1 by overlap PCR (*GCG* indicated replacing Cys with Ala)
PAC/-YF-FPAC/-YF-R	*GAATTC* _ATGGCCAGAACCCTGCAGCTCTCCCTGACTGCTCTCCTCCTGCTG *CCGCGG* GGTGGCCAAGTTGTCGGCCGGGAGGCT	Generation of PAC1, D-PAC1 and M-PAC1 tagged C-terminally with complete YFP. (Restriction sites, *EcoR* I and *Sac*II)
PAC/-Rlu-FPAC/-Rlu-R	*CTCGAG* ATGGCCAGA ACCCTGCAGCTCTCCCTGACTGCTCTC *AAGCTT* GGGTGGCCAAGTTGTCGGCCGGGAGGCT	Generation of PAC1, D-PAC1 and M-PAC1 tagged C-terminally with Rlu. (Restriction sites, *Xho* I and *Hind* III)
YF/N-FYF/N-R	*CCGCGG* ATGGTGAGCAAGGGCGAGGAGCTGTTC *GCGGCCGC* TTA CTCGATGTTGTGGCGGATCTTGAAGTT	Amplification of the YFP N-terminal 172AA and tagging PAC1, D-PAC1 and M-PAC1 C-terminally. (Restriction sites, *Sac* II and *Not* I)
YF/C-FYF/C-R	*CCGCGG* GACGGCAGCGTGCAGCTCGCCGACCAC *GCGGCCGC* TTACTTGTACAGCTCGTCCATGCCGAG	Amplification of the YFP C-terminal 67AA and tagging PAC1, D-PAC1 and M-PAC1 C-terminally. (Restriction sites, *Sac* II and *Not* I)

### Cell Culture and Transfection

Chinese hamster ovary (CHO) cell line CHO-K1, which has been shown to express neither PACAP nor PAC1 [18], was used for the transient and stable expression of receptor constructs. Cells were grown in Dulbecco’s modified Eagle’s medium(DMEM) supplemented with 10% fetal calf serum in a humidified atmosphere of 95% air, 5% CO_2_ at 37°C. The cells were transfected with intact PAC1 or deletion mutant D-PAC1 or Cys mutant M-PAC1 constructs using lipofectamine LTX and Opti-MEM medium (Invitrogen, CA) following the instruction book. For transient expression, 3 ug of total plasmid DNA was typically added into each 10-cm^2^ Petri dish, cells were studied 48 to 72 h after transfection to allow maximal levels of expression of recombinant proteins. For stable expression, cells transfected with plasmids were selected based on G418 (0.8–1 mg/mL) insensitivity, then were cloned by successive cycles of limiting dilution and screened by the fluorescent signal. Cells were maintained in DMEM medium supplemented with 10% FBS and 0.8 mg/mLG418 with an atmosphere of 95% air, 5% CO_2_ at 37°C.

### Fluorescence Confocal Microscopy

Cellular trafficking of receptor constructs were evaluated by visualizing fluorescence in CHO cells expressing YFP-tagged receptor constructs. The transfected cells grown on Petri dish were mounted on microscopic slides. YFP fluorescence was acquired using appropriate spectral settings (excitation, 488 nm argon laser; emission, 545 nm filter; pinhole diameter 2.3 airy units) of the confocal microscope (LSM 510 META; Zeiss, Thornwood, NY) equipped with a Plan-Apochromat63×/1.4 numerical aperture oil objective. For the detection the effects of the oligopeptide HSDCIF on the trafficking of PAC1, live cells transfected with PAC-YFP in the dish were mounted on the microscopic slides, and fluorescent images were collected every 16 s for total 1120 s starting from the time of the addition of the oligopeptide HSDCIF (to a final concentration of 100 nmol/L) on.

### Immunofluorescence

The expression of receptors tagged with the N-terminal fragment of YFP (Y/N) or the C-terminal fragment of YFP (Y/C) was determined by immunofluorescence. Cells expressing the receptor fusion protein were fixed with 4% (w/v) paraformaldehyde (PFA) in PBS for a maximum of 5 minutes at room temperature before being washed twice with PBS and incubated in 3% (w/v) BSA in PBS (blocking solution) for one hour at room temperature. For cells that needed permeabilization (to allow entry of an antibody that recognizes an intracellular epitope, e.g. BXP-21), a 5 minute incubation in 0.05% (v/v) TritonX-100 in PBS was included before the blocking step. Following blocking, cells were then incubated with rabbit polyclonal antibody raised against amino acids 61–115 mapping within an N-terminal extracellular domain of mouse PAC1 (1∶100; Santa Cruz Biotechnology, USA) for 1 h at room temperature and then washed twice with PBS. Cells were then incubated for a further 1 h with an Alexa594-conjugated anti-rabbit antiserum (1∶400). After washing with PBS, cells were viewed using the OLYMPUS converted fluorescence microscope IX71 (Japan) with excitation 520±30 nm and emission 595±30 nm .

### Bimolecular Fluorescence Complementation (BiFC)

The BiFC assay is based on the reconstitution of a fluorescent protein molecule upon re-association of its two nonfluorescent fragments [19]. If YFP was cut into N-terminal (173 amino acid residues) and C-terminal (67 amino acid residues) segments, neither of them displayed fluorescent property when expressed alone. Co-expression of the segments linked to interacting proteins allowed the partial reformation of YFP with the concomitant appearance of the fluorescent signal. Cells were co-transfected with 3 µg of receptor DNA per 10-cm^2^ Petri dish, divided equally among two receptor constructs for each plasmid pair. And 48 hours subsequently, cells were imaged under converted fluorescence microscope using the excitation (480±30 nm) and emission (535±25) filter.

For statistic analysis, assays were performed for aliquots of approximately 25,000 cells per well in 96-well white Optiplates. In brief, the CHO cells were innoculated equably in 96-well plate and transfected with the equal amount plasmids (1.0 ug of DNA/5×10^5^ divided equally into two receptor constructs for each plasmid pair). The fluorescent signals from the cells 48 hours after transfection were detected in Victor3 1420 multi-label counter (PerkinElmer, Wellesley, MA) using the excitation (480±30 nm) and emission (535±40 nm) filter. The cells transfected with D-PAC-YFP or M-PAC-YFP were used as positive control, and the cell without transfection as negative control. The assays for each transfection were repeated at least six times. For examination of the effects of the oligopeptide HSDCIF on the dimerization, the transfected CHO cells were incubated with oligopeptide HSDCIF (100 nmol/L) for 2 h at 4°C before collecting the BiFC signals. All experiments were run with at least four replicates in parallel and repeated six times.

### Bioluminescence Resonance Energy Transfer Assays (BRET)

The CHO cells were innoculated equably in 96-well white OptiPlate and submitted to co-transfection with Rluc-tagged and YFP-tagged receptor constructs. The BRET assay was initiated 48 hours after transfection by adding the cell-permeant Rlu-specific substrate *coelenterazine h* to the cell suspension to yield a final concentration of 5 uM in a 96-well white OptiPlate. Luminescence and YFP fluorescence intensities were quantified in representative aliquots of the same populations of cells utilized in each set of BRET studies. The BRET signal was collected using the Victor3 1420 multi-label counter (PerkinElmer, Wellesley, MA) with emission filter sets for luminescence (460 nm, bandwidth 25 nm) and fluorescence (535 nm, bandwidth 25 nm). The ratio of fluorescence to luminescence emission from the cells transfected with Rlu-tagged receptor construct alone (1.0 ug of DNA/5×10^5^) was considered as background and used for the determination of correct factor (Cf = Em535/Em460), which defined the amount of signal in the acceptor portion that was attributable to donor bioluminescence. The BRET ratio was calculated based on the ratio of fluorescence to luminescence emission using the following formula: (Em535–(Em460×Cf))/Em460.

BRET titration (saturation) experiments were performed to validate the observations in the static BRET studies imitating the method decribed by Harikumar KG [20]. For this, CHO cells were transfected both with a constant amount of donor construct (Rlu-tagged receptor construct at a concentration of 1.0 ug of DNA/5×10^5^ cells) and with increasing amounts of acceptor construct (YFP-tagged receptor construct at concentrations of 0.3 to 6.0 ug of DNA/5×10^5^ cells). The BRET ratios were plotted against the acceptor-to-donor ratios. Curves were fit to these data and were evaluated for quality-of-fit based on R2 values using Prism 3.0. When a single phase exponential curve was found to represent a significantly better fit than the linear function (F test determination with p value<0.05), it was utilized to calculate the BRETmax and BRET50 values. For examination of the effects of the oligopeptide HSDCIF, the transfected CHO cells were incubated with 100 nmol/L for 2 h at 4°C before the BRET signals were collected . All experiments were run with at least four replicates in parallel and repeated six times.

### Western Blotting

In order not to destroy the dimer of PAC1, SDS-PAGE was conducted in non-reducing condition. After mixing the samples with loading buffer without reducing agent and boiling, both cell membrane samples and cell lysates were subjected to SDS-PAGE analysis using 4 to 12% Bis-Tris gels (NuPAGE; Invitrogen) and MOPS buffer. After electrophoresis, proteins were transferred onto nitrocellulose membranes that were incubated in 5% nonfat milk and 0.1% Tween 20/PBS solution at room temperature on a rotating shaker for 2 h to block nonspecific binding sites. The membrane was incubated overnight with a rabbit polyclonal antibody raised against amino acids 61–115 mapping within an N-terminal extracellular domain of mouse PAC1 (Santa Cruz Biotechnology, USA) and detected using a horseradish peroxidase-linked anti-rabbit IgG secondary antiserum (GE Healthcare, Little Chalfont, Buckinghamshire, UK). Immunoblots were developed by the application of enhanced chemiluminescence solution (Pierce Chemical, Rockford, IL).

### Cell Proliferation Assay

To investigate the effects of the oligopeptide HSDCIF and PACAP on the growth of CHO cells expressing PAC1 stably named PAC1-CHO constructed as previously described [21], the cells (2×105 cells/well) were plated in 96-well plates overnight at 37°C in DMEM with 0.5% FBS. The next day the cells were incubated with or without gradient concentrations of HSDCIF or PACAP in the absence of FBS for 24 h. The viability of the cells was determined using a colorimetric MTT assay (Methylthiazoletetrazolium bromide, Sigma). This assay is based on the reduction of MTT into a blue formazan dye by viable mitochondria. At the end of the 24 h treatment, the medium was discarded from the plates, and the cells were subsequently washed twice with PBS. The cells were then incubated with PBS containing 0.5 mg/mL MTT for 4 h at 37°C in an atmosphere of 5% CO_2_. The solution was removed carefully, and 1 mL of dimethylsulfoxide was added to dissolve the blue-colored formazan particles. The samples were transferred to a 96-well plate, and the absorbance was measured using an ELISA reader (Bio-Rad microplate reader, Bio-Rad) at 570 nm, representing the values in arbitrary units (AU). All experiments were run with at least four replicates in parallel and repeated six times, and the results are expressed as a percentage of the control (treatment without any peptides). In order to test the effects of HSDCIF on the activity of PACAP, the cells viability was evaluated in the presence of one dose of HSDCIF peptide (100 nM) with PACAP in gradient quantities (10 to 1000 nM). Moreover, reverse experiment was performed in the presence of one dose of PACAP (100 nM) with various doses of HSDCIF peptide (10 to 1000 nM).

### Statistical Analysis

Statistical analysis was performed with GraphPad Prism, using the unpaired t test. Differences with p <0.05 were considered to be statistically significant.

## Results

### Expression Difference between PAC1 and D-PAC1 in CHO Cells

**Figure 2:**
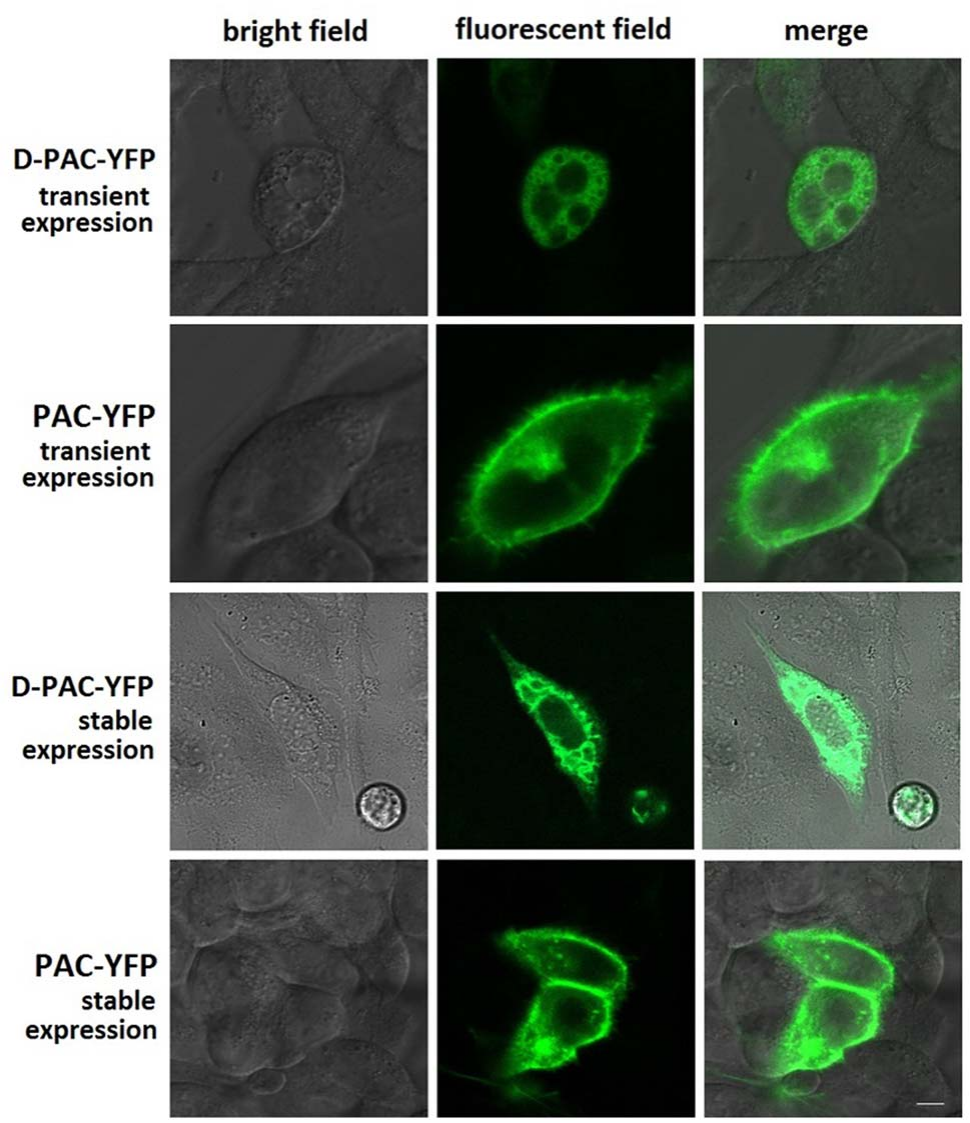
Morphologic localization of YFP-tagged PAC1 and YFP-tagged D-PAC1. Shown were confocal fluorescence micrographs of CHO cells expressing YFP-tagged PAC1 and YFP-tagged D-PAC1 transiently and stably in light field, fluorescent field and their merge. It was observed that in both transient and stable expression, D-PAC1 construct was not able to traffic to cell membrane and accumulated inside the cells,while PAC1 construct was transported to the cell surface normally. Bar, 5 µm.

The results of fluorescent confocal imaging (Fig. 2) showed that PAC1 was normally trafficked to the cell surface when it was expressed transiently or stably in CHO cells. On the contrary, D-PAC1 with the deletion of N-terminal HSDCIF motif was not trafficked to the cell surface and accumulated inside the cells on the endoplasmic reticulum (RE), when no matter D-PAC1 was transiently expressed or stably expressed in CHO cells.

### BiFC Analysis of the Dimerization of PAC1 and D-PAC1

**Figure 3:**
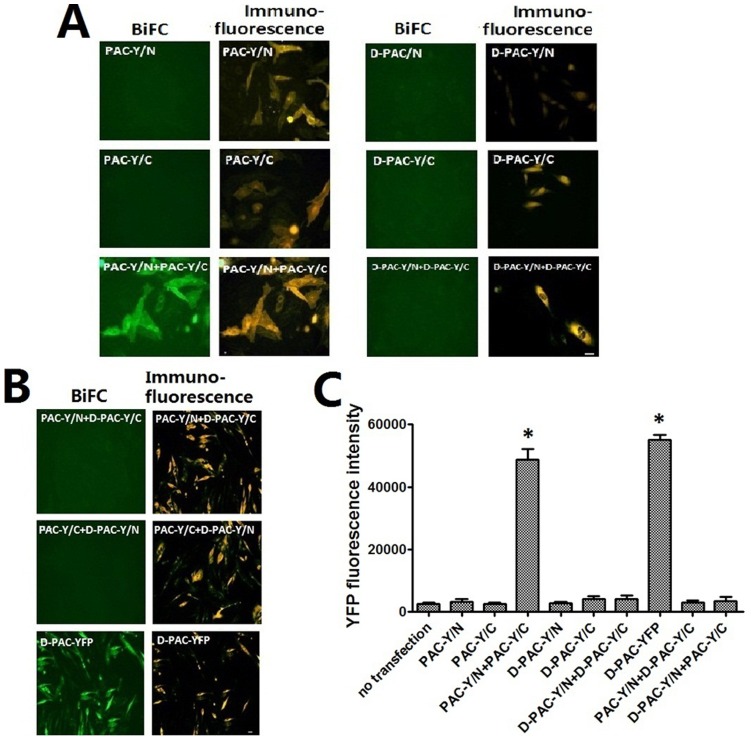
Bimolecular fluorescence complementation and immunofluorescence (A, B). Shown were fluorescence images of CHO cells expressing various receptor constructs, as indicated. The positive results of immunofluorescence using antibody against PAC1 showed that all constructs expressed PAC1 or D-PAC1. Cells transfected with alone Y/N- or Y/C- tagged receptors did not produce YFP fluorescence. YFP fluorescence could be visualized in the cells transfected with the PAC-Y/N +PAC-Y/C supporting homo-dimerization of PAC1. The cells transfected with D-PAC-Y/N +D-PAC-Y/C produced no YFP fluorescence. Absence of fluorescence supported no physical interaction between PAC1 and D-PAC1, while D-PAC-YFP was used as a positive control producing YFP fluorescence. Bar, 5 µm. The statistical analysis of the YFP fluorescence intensity (C) showed that only cells tranfected with PAC-Y/N +PAC-Y/C or D-PAC-YFP (positive control) had significantly higher YFP fluorescence intensity than the negative control (cells without transfection) (*p<0.01 vs. no transfection). Data were presented as means±S.E. of six independent experiments.

The BiFC assay provided the potential for direct visualization of receptor dimerization in living cells. The results of immunefluorescen assay indicated that all receptor constructs were expressed in the CHO cells (Fig. 3). It was shown in Fig. 3A, expression of either PAC-Y/N or PAC-Y/C alone did not produce detectable fluorescence with characteristics typical of intact YFP. And the YFP fluorescence was observed in the cells co-transfected with PAC-Y/N+PAC-Y/C, which indicated the dimerization of intact PAC-1. But as for D-PAC1, co-transfected of D-PAC-Y/N+D-PAC-Y/C did not produce visible YFP fluorescence, which indicated the deletion of N-terminal HSDCIF motif of the receptor resulting in the failure of the receptor dimerization.

In order to determine whether PAC1 and its deletion mutant D-PAC1 form dimer with each other, tow receptor construct pairs: PAC-Y/N+D-PAC-Y/C and PAC-Y/C +D-PAC-Y/N was used to co-transfect the CHO cells. As shown in Fig. 3B, no typical YFP fluorescence was observed indicating no interaction between PAC1 and D-PAC1. D-PAC-YFP was used as positive control.

After the YFP fluorescence intensity under each transfection condition was collected and plotted, it was shown in Fig. 3C that only cells transfected with PAC-Y/N+PAC-Y/C and cells transfected with D-PAC-YFP (positive control) yielded significantly high YFP fluorescence intensity than the un-transfected cells (negative control). These results showed that D-PAC1 could not form homo-dimer and could not form hetero-dimer with PAC1 either.

### BRET Analysis of the Dimerization of PAC1 and D-PAC1

**Figure 4:**
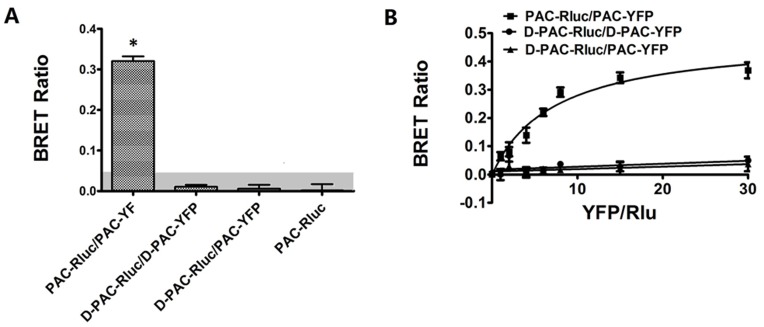
Static BRET assays (A). Shown were BRET ratios generated from CHO cells expressing Rlu-tagged receptor with YFP-tagged receptor constructs as indicated. For static BRET, a total of 1.0 ug of DNA/5×105 divided equally among the noted constructs in each condition was utilized. The shaded area represents the nonspecific BRET signal generated between PAC-Rlu and soluble YFP protein, with BRET signals above this area considered to be significant. Data were presented as means±S.E. of six independent experiments. *p<0.01, significantly above the background and significantly higher than negative control (PAC-Rluc). Saturation BRET assays (B). Shown were the BRET saturation curves plotted as ratios of YFP fluorescence to Rlu luminescence that were observed for tagged receptor constructs studied with a fixed amount of donor and increasing amounts of acceptor. PAC-Rluc/PAC-YFP receptor constructs yielded exponential curves that reached asymptotes indicating significant homo-dimerization of PAC1, while D-PAC-Rluc/D-PAC-YFP and D-PAC-Rluc/ PAC-YFP yielded curves not different from a straight line, indicating that D-PAC1 lost the ability to form dimer with itself and with PAC1. The data are represented as the means±S.E. of six independent experiments.

Bioluminescence resonance transfer was further used to examine whether PAC1 or D-PAC1 homo-dimerized. In this approach, Rlu-tagged receptor was utilized as potential donor and YFP-tagged receptor as acceptor. The static BRET (Fig. 4A) showed that PAC-Rluc/PAC-YFP produced a significant signal, while there was no clear BRET signals in groups D-PAC-Rluc/D-PAC-YFP and D-PAC-Rluc/PAC-YFP similar with the negative control PAC-Rluc. The static BRET results were confirmed further with the saturation BRET experiments (Fig. 4B). Receptor construct pair PAC-Rluc/PAC-YFP yielded a saturable signal reaching an asymptote, while the pairs of D-PAC-Rluc/D-PAC-YFP and D-PAC-Rluc/PAC-YFP only generated a linear signal reflecting non-specific bystander interactions [20]. The results of BRET were consistent with the results of BiFC.

### The Effects of Oligopeptide HSDCIF on the Dimerization of PAC1

In view of above BiFC and BRET results, D-PAC1 with the deletion of the N-terminal HSDCIF motif not only couldn’t traffic to the plasma membrane, but also couldn’t dimerize. Based on these findings, we hypothesized that a chemically synthesized oligopeptide HSDCIF might exert effects on the dimerization and the trafficking of PAC1.

**Figure 5:**
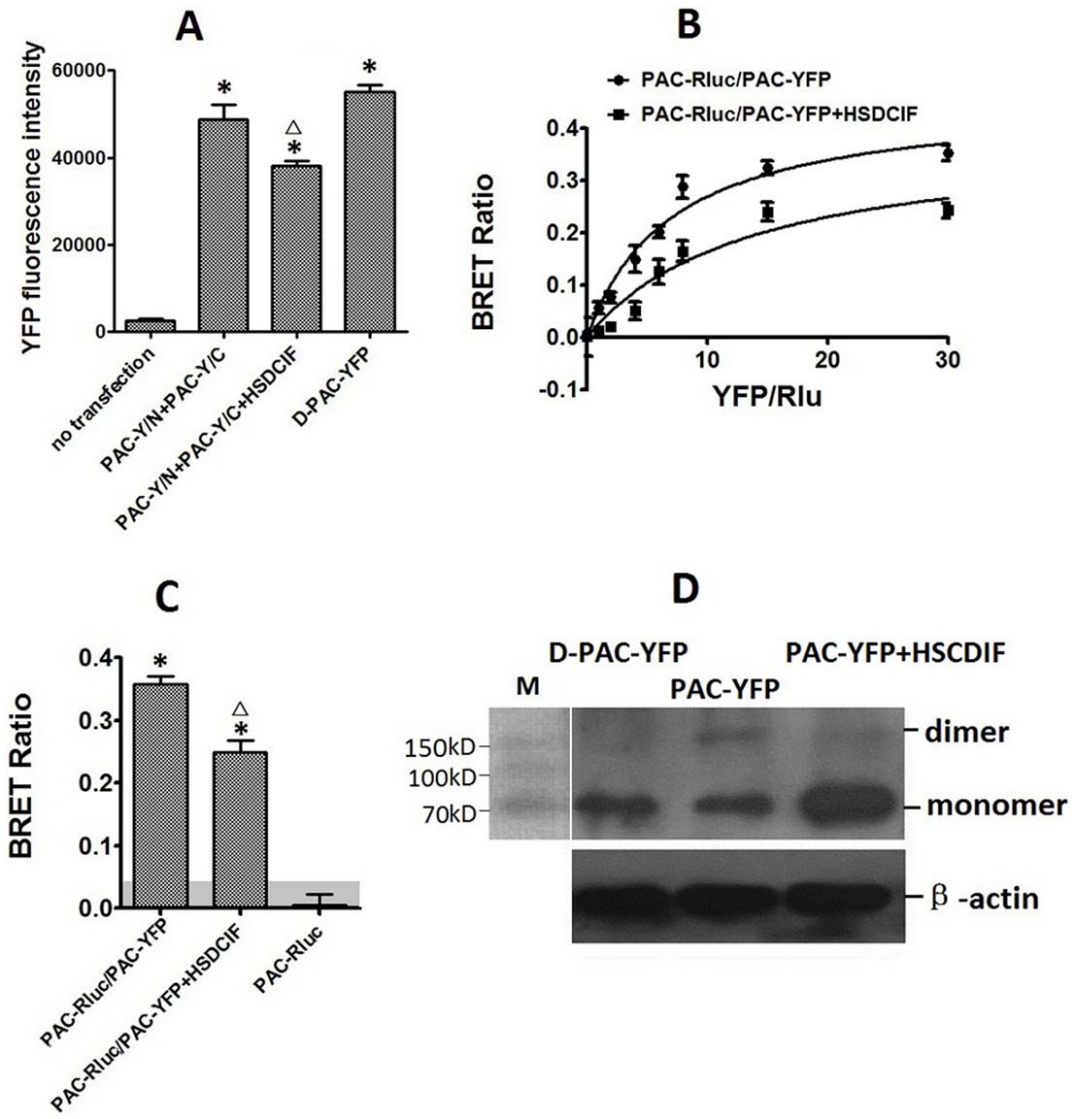
Effects of the oligopeptide HSDCIF on PAC1 BiFC (A). Shown were YFP fluorescence intensity produced by the transfection of the receptor constructs as indicated. The cells without transfection were used as negative control and the cells transfected with D-PAC-YFP as positive control. Exogenous HSDCIF decreased the YFP fluorescence intensity produced by PAC-Y/N+PAC-Y/C significantly. (Δ, p<0.01 vs. PAC-Y/N+PAC-Y/C. * p<0.01 vs. negative control). Data were presented as means±S.E. of six independent experiments. Effects of the oligopeptide HSDCIF on PAC1 saturation BRET (B). Shown were the BRET saturation curves plotted as a ratio of YFP fluorescence to Rlu luminescence that were obtained for pairs of PAC-Rluc and PAC-YFP studied with a fixed amount of donor (1.0 µg of DNA/dish) and increasing amounts of acceptor (0.3–6 µg of DNA/dish). The experiments were performed in the absence or presence of the oligopeptide HSDCIF. PAC1 produced a significant saturable exponential curve, while incubation with HSDCIF lowered the curves significantly. The data are represented as the means±S.E. of six independent experiments. Effects of the oligopeptide HSDCIF on PAC1 static BRET (C). BRET ratios for CHO cells expressing receptor constructs as indicated. The shaded area represents the nonspecific BRET signal generated between PAC-Rlu and soluble YFP protein, with BRET signals above this area considered to be significant. *,p<0.01, significantly above the background and significantly higher than negative control (PAC-Rluc). The BRET ratio in PAC1-expressing CHO cells incubated with HSDCIF was significantly lower than that in cells without treatment with HSDCIF (Δ, p<0.01 vs. PAC-Rluc/PAC-YFP). The data are presented as the means±S.E. of six independent experiments. Westernblot analysis of CHO cells expresing PAC-YFP, D-PAC-YFP, and PAC-YFP expressing cells incubated with exogenous oligopeptide HSDCIF (D). As shown, the band with the molecular weight (about 160 kD) consistent with the molecular weight of the dimer was absent in D-PAC-CHO, and the exogenous oligopeptide HSDCIF decreased the dimer amount in PAC-CHO significantly.

**Figure 6:**
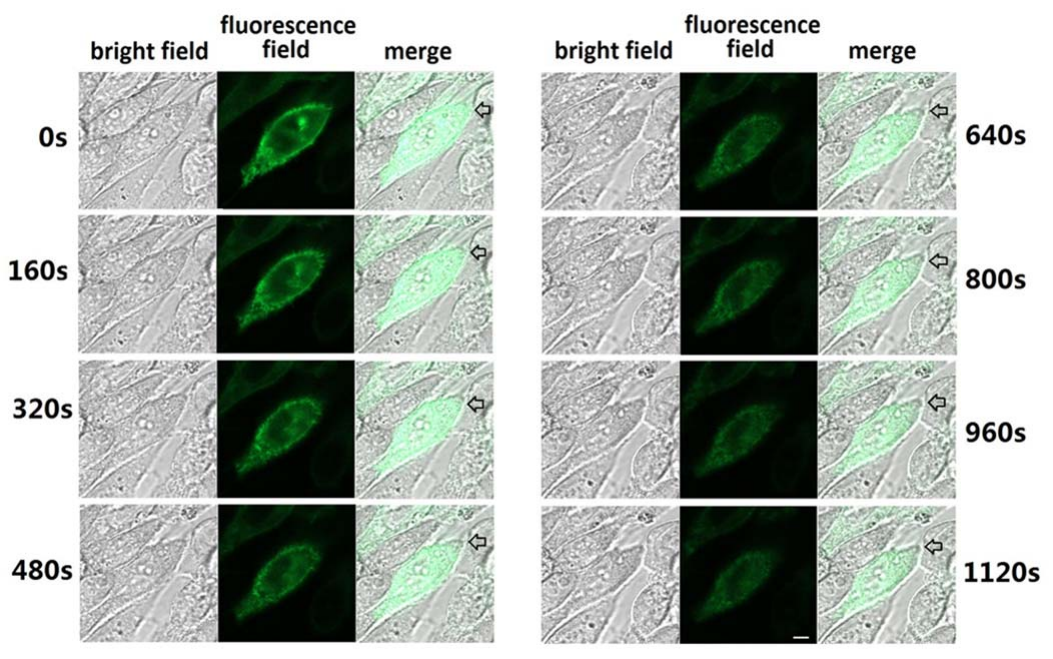
Effects of oligopeptide HSDCIF on the traffic of PAC1. Eight fluorescence confocal images from the animation of 0–1120 s after the addition of oligopeptide indicated that the fluorescence presenting the site of PAC1 detached from the cell-surface and moved to the inside of the cell, especially significantly in the region shown by the arrow. Bar, 5 µm.

BiFC, BRET and westerblot were used to detect the effects of the oligopeptide on the dimerization of PAC1. The transfected CHO cells were incubated with 100 nmol/L HSDCIF for 2 h at 4°C before the signals of BiFC and BRET were collected and westernblot was perform. As shown in Fig. 5A, the statistical analysis of BiFC showed that HSDCIF (100 nmol/L) significantly reduced the YFP fluorescence intensity produced by PAC-Y/N+PAC-Y/C (p<0.01). And both static and saturation BRET (Fig. 6B, C) showed that 100 nmol/L HSDCIF significantly decreased the BRET ratio produced by PAC1 dimerization (p<0.01). The results of westernblot showed that the band with the molecular weight about 160 kD consistent with the molecular weight of the dimer is absent in the cells expressing D-PAC-YFP, while the incubation of exogenous oligopeptide HSDCIF reduced the amount of the dimer in the cells expressing PAC-YFP significantly. All these results indicated that oligopeptide HSDCIF could inhibit the dimerization of PAC1.

### The Effects of Oligopeptide HSDCIF on the Trafficking of PAC1

Live cells 48 h after transfection with PAC-YFP was subject to fluorescent confocal image. Images were collected every 16 s and the animation was made starting from the time point when the oligopeptide HSDCIF was added into the cells to final concentration of 100 nmol/L. Eight images from 0–1120 s of the animation in Fig. 6 showed that the fluorescence presenting the location of PAC1 aggregated on the cell-surface at initial time (0 s) and was found detached from the cell-surface 160 s after the addition of the oligopeptide. And as time went on, the fluorescence located on the cells surface broke away and more and more fluorescence moved from the cell surface into the intracellular region. The addition of oligopeptide HSDCIF induced the internalization of PAC1.

### Cell Trafficking and Dimerization of M-PAC1 Replacing Cys with Ala

**Figure 7:**
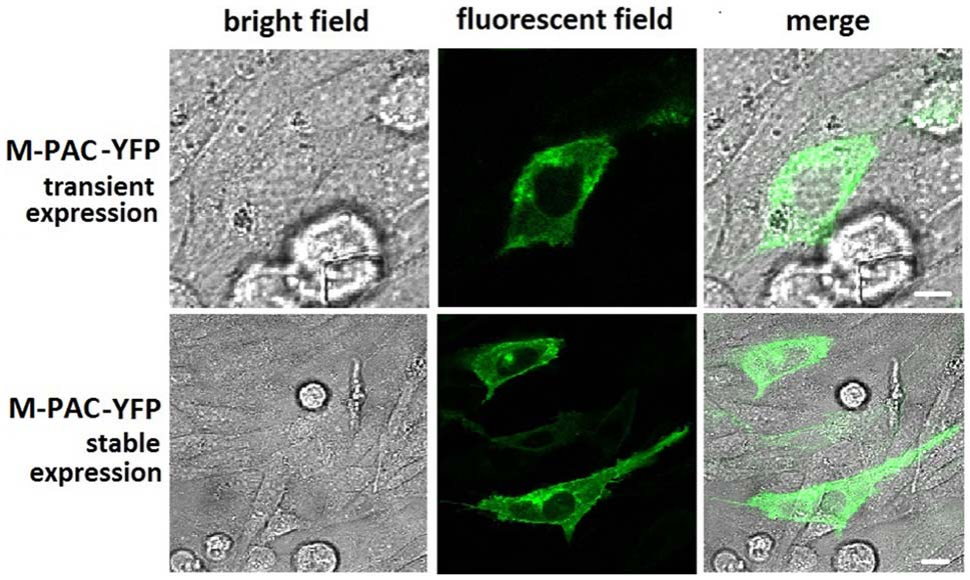
Morphologic localization of YFP-tagged M-PAC1. Shown were confocal fluorescence micrographs of CHO cells expressing YFP-tagged M-PAC1 transiently and stably in light field, fluorescent field and their merge. It was observed that in both transient and stable expression,M-PAC1 construct was transported to the cell surface normally. Bar, 5 µm.

**Figure 8:**
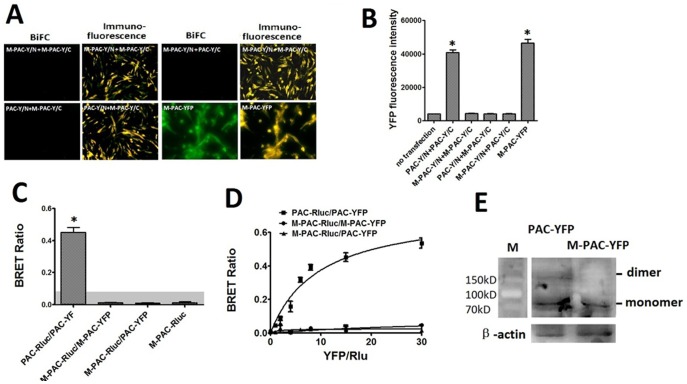
BiFC and immunofluorescence (A). Shown were fluorescence images of CHO cells expressing various receptor constructs, as indicated. The positive results of immunofluorescence using antibody against PAC1 showed that all constructs expressed M-PAC1. The cells transfected with M-PAC-Y/N +M-PAC-Y/C produced no YFP fluorescence. Absence of fluorescence supported no physical interaction between PAC1 and M-PAC1, while M-PAC-YFP was used as a positive control producing YFP fluorescence. Bar, 5 µm. The statistical analysis of the YFP fluorescence intensity (B) usied M-PAC-YFP as positive control and cells without transfection as the negative control (*p<0.01 vs. no transfection). Data were presented as means±S.E. of six independent experiments. Static BRET assays (C). Shown were BRET ratios generated from CHO cells expressing Rlu-tagged receptor with YFP-tagged receptor constructs as indicated. The shaded area represents the nonspecific BRET signal generated between PAC-Rlu and soluble YFP protein, with BRET signals above this area considered to be significant. Data were presented as means±S.E. of six independent experiments. *p<0.01, significantly above the background and significantly higher than negative control (PAC-Rluc). Saturation BRET assays (D). Shown were the BRET saturation curves plotted as ratios of YFP fluorescence to Rlu luminescence that were observed for tagged receptor constructs studied with a fixed amount of donor and increasing amounts of acceptor. M-PAC-Rluc/M-PAC-YFP and M-PAC-Rluc/ PAC-YFP yielded curves not different from a straight line, indicating that M-PAC1 lost the ability to form dimer with itself and with intact PAC1. The data are represented as the means±S.E. of six independent experiments. Westernblot analysis of CHO cells expresing PAC-YFP and M-PAC-YFP (E). As shown, the band with the molecular weight (about 160 kD) consistent with the molecular weight of the dimer was absent in M-PAC-CHO.

Since it was found that the N-terminal HSDCIF motif was essential for the cell trafficking and the dimerization of PAC1, the Cys residue in HSDCIF motif maybe play a key role, because it is the only amino acid that differed from PACAP (1–6) and it’s a single Cys that does not form known intra-molecular disulfide bond. In order to determine the role of the Cys in the cell trafficking and the dimerization of PAC1, we replaced the N-terminal Cys with Ala to construct a mutant PAC1 named M-PAC1. The fluorescent confocal imaging (Fig. 7) showed that M-PAC1 was normally trafficked to the cell surface when it was expressed transiently or stably in CHO cells. The BiFC, BRET and western blot showed that M-PAC1 lost the ability to form dimer not only with itself but also with native PAC1 (Fig. 8), which indicated that it was the Cys in N-terminal HSDCIF motif that mediated the dimerization of PAC1.

### Effects of Oligopeptide HSDCIF on Viability of the PAC1-CHO Cells

**Figure 9:**
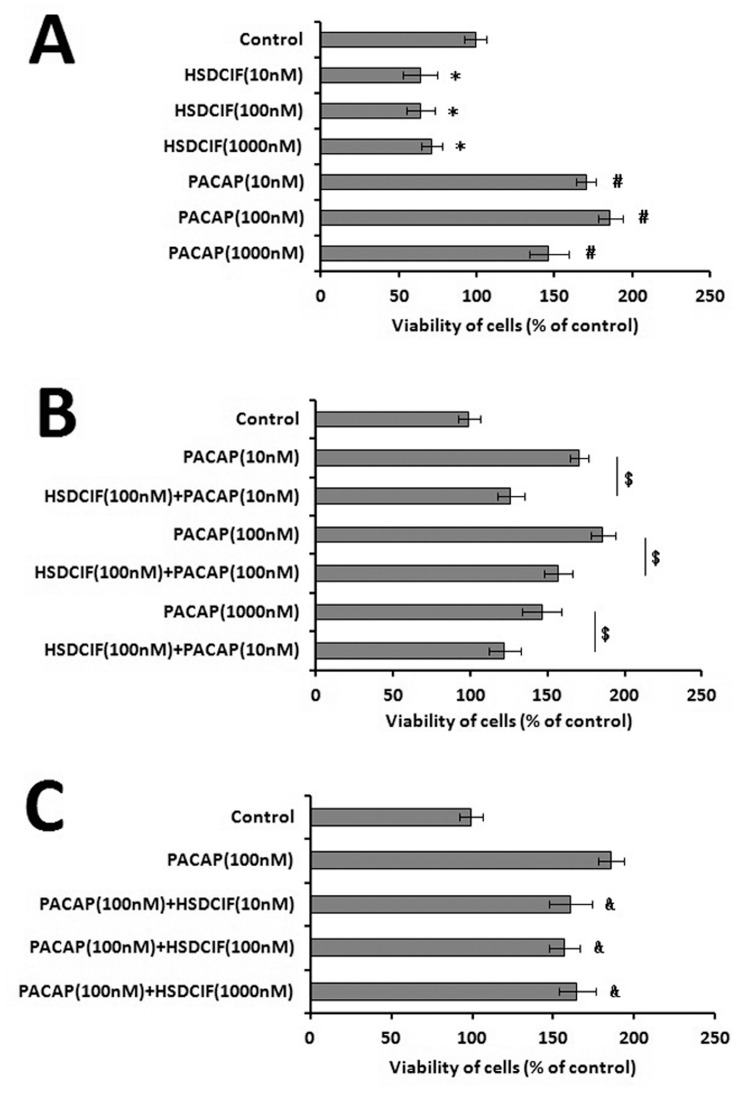
Effects of oligopeptide HSDCIF on the viability of PAC1-CHO cells as measured by MTT assay (A). Data are presented as means ±S.E. obtained from six independent experiments. *p< 0.01 HSDCIF groups vs. control group, #p< 0.01, PACAP groups vs. control group. As shown, exogenous HSDCIF had contrary effects with PACAP on the cell viability. Effects of oligopeptide HSDCIF (100 nM) on the activity of PACAP with gradient concentrations (B). Data are presented as means ±S.E. obtained from six independent experiments. $p< 0.01 HSDCIF(100 nM)+PACAP groups vs. PACAP groups. Effects of oligopeptide HSDCIF with gradient concentrations on the activity of PACAP(100 nM) (C). Data are presented as means ±S.E. obtained from six independent experiments. &p< 0.01 HSDCIF+PACAP(100 nM) groups vs. PACAP(100 nM) group. As shown, exogenous HSDCIF decreased the effects of PACAP on the cell viability significantly.

The effect of the oligopeptide HSDCIF on the growth of PAC1-CHO cells was investigated using the MTT assay. Incubation of PAC1-CHO cells with 10–100 nmol/L HSDCIF in absence of FBS for 24 h led to the significant decreases in the number of viable cells (Fig. 9 A) to about 65% of control, while 10–100 nmol/L PACAP increased the viability of the cells significantly to over 150% of the untreated cells. The results indicated that the cell signals induced by HSDCIF were different from that induced by PACAP.

To test the effects of HSDCIF on the activity of PACAP, the cell viabilities promoted by PACAP with gradient concentrations and a fixed dose (100 nM) of HSDCIF and the cell viabilities affected by oligopeptide HSDCIF with gradient concentrations and a fixed dose (100 nM) of PACAP were assayed. It was found that (Fig. 9B, C) HSDCIF decreased the activity of PACAP on the cell viability significantly (p<0.01) maybe due to its effects on the status of PAC1 including de-dimerization and internalization.

## Discussion

As specific receptor for PACAP, PAC1 is abundantly located in the central nervous system (CNS) and peripheral nervous system,which mediates the most effects of PACAP as neurotransmitter, neuro-modulator, neurotrophic factor, and neuro-protector. PAC1 is currently considered as a potential target for the treatment of neurodegenerative and neuropathic diseases [22]. The high expression of PAC1 has also been reported in some specific physiological and pathological status. Case in point, the expression of PAC1 is increased in neuroendocrine tumors, in the aged rat brains and the human brains suffer from post-traumatic stress disorder [23], [24], [25]. The pharmacological and functional consequences of the up-regulation of PAC1 expression remain incompletely understood. This paper reports several interesting findings about PAC1 for the first time: 1) The N-terminal HSDCIF motif is essential for the cell-surface trafficking of PAC1; 2) PAC1 can form dimer; 3) The Cys residue in HSDCIF motif is crucial for the dimerization of PAC1. These findings may help us to understand the physiological and pathological role of PAC1 in more detail.

Till now, little is known about the mechanism of the cell membrane translocation of PAC1. And it is firstly reported that the N-terminal HSDCIF motif is crucial for the cell surface trafficking of PAC1. Although it has been reported that the N-terminal signal sequence (1–30AA) of VPAC1, another receptor for PACAP and also a member of class B GPCR, is crucial for the receptor expression at the cell surface [26], the HSDCIF motif is not included in the putative signal sequence (1–20AA) of PAC1, and contains no classic N-glycosylation residue Asn, which is involved in glycosylation-dependent cell surface expression of VPAC1 [27]. Furthermore, in this study the signal sequence (1–20AA) was retained in the deletion mutant D-PAC1, which was found not sufficient for the surface trafficking of PAC1. These results showed that the mechanism for PAC1 cell surface delivery might be different from that for VPAC1. The replacement of Cys in the HSDCIF motif with Ala did not affect cell surface trafficking of PAC1 indicating that the other residues in the HSDCIF motif except Cys may be crucial for cell surface trafficking. The HSDCIF motif is just located behind the signal sequence, and contains a charged residue Asp. The charged extra cellular residue Asp was shown to be crucial for the cell-surface expression of GPCR for neurohypophysial hormone vasopressin and oxytocin [28]. These characteristics are consistent with its key role in the cell-surface trafficking. The molecular basis involving the HSDCIF motif for the cell surface trafficking of PAC1 needs more detailed investigation.

Although the dimerization of PAC1 can be deduced by the oligomerization of intra-family member VPAC1, VPAC2 and Secretin receptor [10], [11], [12], no other experimental evidence till now support the dimerization of PAC1 except the results of BiFC and BRET shown in this article. In the oligomerization of Secretin receptor, transmembrane (TM) segment IV was shown as the determinant for oligomerization [20]. It was shown for the first time that the N-terminal Cys in HSDCIF motif was crucial for the dimerization of PAC1. The residue cystein (Cys) in the HSDCIF motif is the first single Cys of PAC1, not be included in the conserved intra-molecular three disulfides in the N-terminal extracellular segment of class B GPCR [29], [30]. The intermolecular disulfide bond may be form through this Cys, which may be the reason why the exogenous oligopeptide HSDCIF competitively inhibited the dimerization of PAC1. Similar findings have been reported in many GPCR. A case in point, the intermolecular disulfide has been shown to contribute to the dimerization of metabotropic glutamate receptor 5 [31], [32]. Moreover, the intermolecular disulfide bond through the Cys in the HSDCIF motif maybe contribute to the interaction of PAC1 not only with itself termed homodimerization, but also with other GPCR termed heterodimerization, or with accessory protein such as receptor activity modifying proteins (RAMP). The putative interactions of PAC1 with other macro molecules through this Cys in the HSDCIF motif need more exploration.

The HSDCIF motif has high homology with PACAP (1–6) (HSDGIF) (shown in Fig. 1C), which is responsible for the activation of PAC1 [15], [16]. Our previous data showed that the cyclo-peptide *CHSDGIC* from the cyclization of PACAP (1–5) with disulfide had potent activity towards PAC1 at the work concentration of 10–100 µmol/L [33]. But it was shown in this article that contrary to PACAP, the exogenous oligopeptide HSDCIF inhibited the proliferation of CHO cells expressing PAC1 at the working concentration of 100 nmol/L combined with the internalization and the de-dimerization of PAC1. Furthermore, exogenous oligopeptide HSDCIF decreased the activity of PACAP of promoting cell viability. A similar finding contradictory to most studies showing PACAP increased cell survival reported that PACAP enhanced the oxidative stress induced cell death of human choriocarcinoma cells [34]. As we known, the status of receptor such as externalization or internalization, oligmerization or not can alter the cells signals and the physiological functions of GPCR [6], [7], [8], [9]. The cell signals and consequent function induced by the exogenous oligopeptide HSDCIF were obviously different from that induced by PACAP. In our opinion, the changes of the receptor status including internalization and de-polymerization induced by exogenous oligopeptide HSDCIF may in turn change the sensitivity of PAC1 to its agonist and the consequent cell signals after receptor activation, which may be the reason why the oligopeptide HSDCIF showed contrary effects to PACAP and decreased the activity of PACAP.

All in a word, this paper showed that the N-terminal HSDCIF motif played crucial role in the cell surface trafficking and the dimerization of PAC1, the Cys residue in the HSDCIF motif is critical for the dimerization of PAC1 and the exogenous oligopeptide HSDCIF had significant effects on cell signaling, trafficking and the dimerization of PAC1. All these may help us to step forward to the clear annotation of the physiological and pharmacological role of PAC1.
